# Natural Gums as Oleogelators

**DOI:** 10.3390/ijms222312977

**Published:** 2021-11-30

**Authors:** Karol Banaś, Joanna Harasym

**Affiliations:** 1 Department of Biotechnology and Food Analysis, Wrocław University of Economics and Business, Komandorska 118/120, 53-345 Wrocław, Poland; joanna.harasym@ue.wroc.pl; 2 Adaptive Food Systems Accelerator–Science Centre, Wrocław University of Economics and Business, Komandorska 118/120, 53-345 Wrocław, Poland

**Keywords:** natural gums, oleogels, organogels, polysaccharides, emulsion

## Abstract

The natural gums used as high molecular weight oleogelators are mainly polysaccharides that deliver a broad spectrum of possible utilization methods when structuring liquid fats to solid forms. The review discusses a natural gums’ structuring and gelling behavior to capture the oil droplets and form the water/oil gelling emulsions basing on their structural conformation, internal charge, and polymeric characteristics. The specific parameters and characteristics of natural gums based oleogels are also discussed. In the future, oleogels may eliminate saturated and trans fats from food products and allow the production of low-fat products, thus reducing the environmental damage caused by the excessive use of palm oil. The increasing knowledge of molecular interaction in polysaccharide chains of natural gums allows to apply more sustainable and wiser strategies towards product formulation. Innovative solutions for using oleogels based on natural polysaccharide biopolymers let incorporate them into the food matrix and replace fats completely or create blends containing the source of fats and the addition of the oleogel. The profound insight into molecular characteristics of natural gums in the function of being oleogelators is presented.

## 1. Introduction

Fat is one of the essential components of food products, playing a lot of critical technological functions. It affects its sensory characteristics, such as taste and aroma, and textural and physical properties [1,2,3]. The food industry most commonly uses naturally solid or modified liquid fats, which contain unsaturated or saturated fatty acids of trans configuration [4]. Fatty acid isomers of trans configuration have a beneficial effect on the texture of fats, but when consumed in higher amounts, they contribute to cardiovascular disease and metabolic disorders [5]. 

Different methods are used in food production to convert liquid oils into solid fats, like hydrogenation, fractionation, or interesterification [2]. The most commonly used method is the hydrogenation of unsaturated fatty acids of vegetable oils, which produces saturated solid fats characterized by improved oxidative stability and plasticity. This process, although technologically advantageous, carries the risk of creating the trans fatty acids negatively impacting on human health. 

Interestrification, which significantly reduces the content of trans fatty acids in modified fats, cannot fully eliminate them, translating into health problems for consumers [6]. Conversely, removing the saturated acids would result in abnormal physical and sensory changes in typical product that were unacceptable to consumers [2,7]. 

An alternative solution to the previously mentioned problems is the concept of oleogels in food production. Oleogelation may become a promising method in the future for curing liquid fats rich in unsaturated fatty acids. This process can change the consistency of liquid vegetable or fish oils and give them the properties of solid fats, without the participation and presence of harmful saturated trans fatty acids in their composition. 

**Figure 1:**
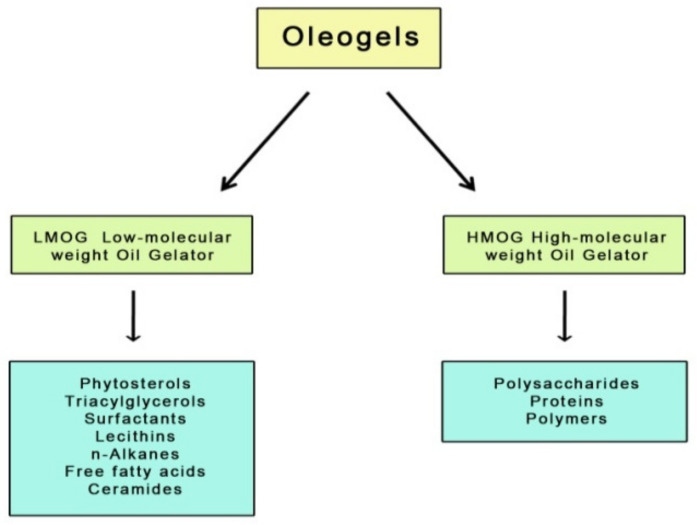
Gelator compounds of different molecular weight.

The oleogelation method involves creating a semi-solid oil form using gelling substances [8]. Oil structuring is based on the physical transformation of dissolved gelators in a lipid environment whose chemical characteristics are constant during the process [9]. Gelators facilitating solid structure development can be divided into two groups; these are low molecular weight oil gelators (LMOG) and high molecular weight oil gelators (HMOG) (Figure 1). 

The first group refers to small molecules that build a fixed crystalline network stabilizing the oil phase through self-organization. This process occurs under controlled temperature conditions and is driven by existing physical interactions between the molecules, such as hydrogen bonds, hydrophobic bonds, and Van der Waals interactions. Due to the formation mechanism and physical interactions, the structure of these oleogels is susceptible to shear forces and temperature [10]. 

LMOGs include, for example, naturally occurring simple sugars (e.g., trehalose, sucrose, mannose, amygdaline), glycosides (raspberry glycoside ketone, salicin), and sugar alcohols (e.g., xylitol, sorbitol, mannitol) [11], but also waxes, fatty acids, carbamates, lecithins, ceramides, monoacylglycerols, diacylglycerols, triacylglycerols, n-alkanes, and mixtures of γ-oryzanol and phytosterols [12,13]. 

The second group of gelators (HMOGs) includes macromolecular systems capable of forming three-dimensional networks linked to each other by physical interactions such as hydrogen bonds. Considering the polymeric properties of these networks, oleogels built with such gelators have better viscoelastic properties, which are determined by the concentration, molecular weight, and conformation of the polymer [10]. High molecular weight gelling agents used for oleogels include proteins (e.g., β-lactoglobulin) [14,15], some polymers [16] and polysaccharides (e.g., ethylcellulose, hydroxypropyl methylcellulose, alginates) [17,18]. 

Polymers that are most commonly used to form oleogels and are also the most promising group are polysaccharides. These complex sugars widely found in various living organisms perform a wide range of functional properties, including energy storage, support cell and tissue function, and are responsible for cell signaling. The structure of the polymer chain is based on monosaccharide units such as fructose and glucose, which come together via a glycosidic bond during the dehydration reaction [19]. 

The functionality of polysaccharides is a direct result of their molecular structure, which, depending on the type of monosaccharide, the position, and type of glycosidic bonding, can take both linear and branched forms. Hydroxyl groups (-OH) and other side groups of polymer chain structure influence the spatial configuration of the molecule, which directly affects its ability to form intermolecular hydrogen bonds and intramolecular interactions between chains and other elements. In turn, these features lead to higher-order spatial structures, such as the helix [20]. 

An example of a polysaccharide composed of two types of monomers (D-mannuronic acid and L-guluronic acid linked by a β-1,4-glycosidic bond) is alginate originating from seaweed. The structure of alginate results from the arrangement of the individual monomer units and the length of the blocks, which in turn depend on the type of tissue, species, and place of origin of the algae and significantly determine its characteristics as a gelator. The main advantage of using this polysaccharide in oleogelation is the simplicity and low cost of the process and the high mechanical stability and non-toxicity of the resulting gels [21,22].

The approach for using the minimally processed natural gum as oleogelators is worth exploring while research reports considering their specific usage are still scarce despite a variety of existing natural gums. The main explored polysaccharides are still cellulose derivatives. However, they are usually modified physically (crystalline or micro cellulose) or chemically (ethylcellulose). 

We summarize the existing research attempts exploring the possibility of natural gum exploitation as HMOG for oleogels manufacturing. Either in binary or ternary blends with proteins, natural gums offer a very sustainable and ”clean-label” approach for physical oil structuring. And taking into account their systematic classification as fiber ingredients, the resulting oleogels may also offer nutritional benefits beyond the structuring features.

## 2. Oleogelation with Natural Gums

**Figure 2:**
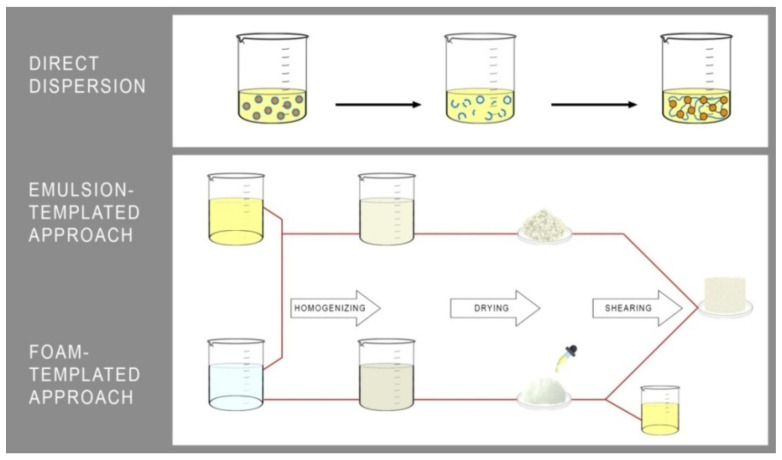
The leading strategies used for oleogels manufacturing.

Gelators (biopolymers), e.g., proteins and polysaccharides used to structure oils, are mostly commercially available, relatively inexpensive, and mostly permitted for use in the food industry [23,24,25]. Among the methods leading to the oleogels formation, three leading strategies stand out, which are: (1) a direct dispersion of structuring agents [26] and the indirect ones based on the formation of (2) structural emulsions [23] or (3) porous foams filled by the liquid oil [27,28] (Figure 2). 

Recently, the increasing interest in using proteins and stabilizing them with polysaccharides as components to form oleogels appeared [29]. Various polysaccharides are used to stabilize such lipid structures like pectin, sodium alginate, xanthan gum, tara gum, gellan gum, acacia gum, and carob gum. These polysaccharides originate from a variety of sources, i.e., carrageenan, alginate derive from algae, while gellan and xanthan gum are of bacterial origin. Biopolymers such as pectin, tara, carob, and acacia gums are of plant derivatives, while chitin comes usually from crustaceans or fungi [30]. 

The molecular structure of individual polysaccharides differs a lot, e.g., alginate, gellan gum, and carrageenan have a linear structure, xanthan, acacia gum, and pectins are hetero-polysaccharides and thus composed of different monomers, while tara and carob gum are galactomannans [31,32].

The molecular structure of gelators significantly affects the physical, chemical, and rheological properties of the resulting oleogel, which is related to the accessibility of the molecule fragments that form the contact sites. Wijaya et al. (2019, 2017) [17,29] proposed an emulsion method for oil structuring using proteins and polysaccharides while also investigating the effect of pH on the properties and stability of oleogels. 

During the lipid structuring, in a first step, water–oil emulsions (from 60% to 80% oil by volume in the emulsion) were prepared stabilized by a matrix consisting of a combination of suitable gelators, in which water was then disposed of during heat treatment to obtain a solid oleogel texture with an increased oil content (above 90%). Studies have shown that using proteins and polysaccharides as gelators results in higher stability of oleogels and slows down the oil release due to their emulsifying and stabilizing properties. However, this depends mainly on the chemical and structural features of the used gelator [16,17,33].

Vélez-Erazo et al. (2020) [30] demonstrated that the most stable oleogels after four weeks of storage at 5 °C were structures that based on xanthan gum and tara gum. These formulations had a uniform and creamy appearance throughout the storage period and showed minimal oil loss. Such behavior was due to the properties of these gelators, which have a high viscosity despite having weaker gelling properties than the other polysaccharides used in the experiment. Water–oil emulsions undergo dehydration during mild drying. The resulting polymer network serves as a building block for the dry product, which, through shearing, gives the final oleogel with the desired texture and functional properties [30]. 

Some studies show that the ideal way to improve the stability of emulsions is to force the polymers to form colloid complexes. Interactions occurring in such an emulsion between, e.g., polysaccharides and proteins and at the water–oil interface result in better hydration, surface properties (i.e., adsorption, charge, surface tension and film thickness), and a more stable structure. By creating nanocomplexes of gelatin, tannic acid, and linseed gum, Qiu et al. (2018) obtained an oleogel made of the three components using an emulsion method [34]. 

The applied tannic acid, which is a derivative of polyphenols, as a natural antioxidant having a large number of hydroxyl groups, interacts with carbonyl groups of polysaccharides and proteins, creating strong covalent and non-covalent interactions. Due to its ability to scavenge, free radicals perform antioxidant functions, thus preventing the oxidation of unsaturated fatty acids in the oleogel. Complexation with tannic acid allows it to accumulate on the surface of the oil droplet and act as a natural crosslinking agent that can manipulate the architecture and improve the antioxidant activity of the emulsion. In addition, the use of linseed gum (an anionic polysaccharide extracted from linseed), characterized by its excellent thickening and emulsifying capacity with good gelling properties, allowed for a more stable oleogel structure (without significant oil escape) with important health benefits [34].

Furthermore, oleogels based on these complexes were characterized by high thixotropy and hydration. The compact structure of the oleogel was made possible by interfacial adsorbed particles on the inner surface of the oil–water phases (this mechanism resembles the stabilization of Pickering emulsion particles in oil–water systems), which formed an interfacial “coating” and also polymer networks. In the future, oleogels based on these gelators may have applications for the production of foods with controlled rheological and textural properties [34]. 

Another method, used as frequently as the emulsion-based one, is based on first producing a porous foam of biopolymers and then saturating the foam with oil and shearing the resulting product with high-energy homogenisation. 

The method proposed by Abdollahi et al. (2020) [28] showed that the addition of xanthan gum increased the viscosity and stability of the foam. Increasing the concentration of xanthan gum multiplied the network density and the hardness, but did not affect the moisture sorption. The oil binding capacity of such oleogels was >92%. The oleogel network produced by the “foam” method can protect edible oil from oxidation during two-month storage. The formation procedure, which makes foam-type oleogels that maintain oil even at high temperatures, may be of interest to researchers looking for solid fat substitutes in food, e.g., in pastry products [28]. 

## 3. Natural Gums Molecular Features Facilitating Oleogelation

**Figure 3:**
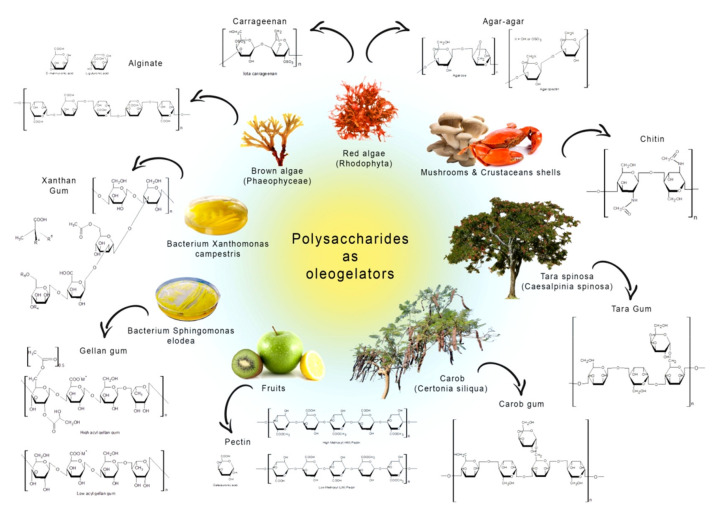
Natural gums structural conformation and origins.

Resins and gums are used mainly as thickening agents in cosmetics, pharmaceutical products, and foodstuffs. They are usually extracts or isolates, sometimes even less purified fractions obtained after water extraction, making them perceived by the consumer as natural and friendly (Figure 3).

### 3.1. Xanthan Gum 

An example of gum that is most commonly used as a thickener in the food industry is xanthan gum produced by the bacteria *Xanthomonas campestris*. It is a high molecular weight heteropolysaccharide (hydrocolloid) composed of D-glucose units forming the cellulose main polymer chain and the side chains diverging from it. The divergent side chains are formed by glucose residues with a trisaccharide chain consisting of two mannose units separated by D-glucuronic acid. The terminal mannose units may have a purine group attached (C-6 carbon), while the inner units may have an acetyl group attached (C-6 carbon). Aqueous solutions of xanthan gum form a pseudo gel structure [35].

Xanthan gum is widely used as a stabilizer in water–oil emulsions and is a widely used non-gelling biopolymer that increases viscosity at zero shear, even at low concentrations. It can be successfully used in the synthesis of oleogels by the emulsion method. As mentioned above, in this method, oleogels are formed by stabilization of an oil-in-water emulsion by surface-active polysaccharides (cellulose derivatives, e.g., MC, HPMC), proteins (gelatin), or surface-inactive polysaccharides (e.g., xanthan gum). During selective water evaporation, oil droplets’ coalescence can occur, resulting in phase separation [36]. 

Xanthan gum has been used to stabilize the polymeric network is an oleogel composed of sunflower oil, gelatin and xanthan gum [16]. Gelatin and xanthan gum formed the aqueous phase, stabilized the emulsion using interfacial adsorption, and increased viscosity in the bulk phase, which translates into a stiffening of the whole network and the formation of interfacial membranes providing better resistance of oil droplets to stresses (dehydration, heat treatment, freezing), compared to emulsions stabilized by monolayer membranes. The research proved that xanthan gum and gelatin interact with each other through hydrophobic interactions and weak electrostatic interactions between carboxyl groups of gum and peptide bond groups of gelatin. This phenomenon is confirmed by spectroscopic results proving shifts in the vibrational bands of the OH and NH groups of the gelatin and xanthan gum coacervate [16,37,38]. Patel et al. (2015) also confirmed that the coexistence of these two components, i.e., gelatin and xanthan gum, guarantees more excellent stability of the oleogel during the drying stage, which can usually result in phase separation and separation of the oil from the system, as confirmed by the case of an emulsion formed with the addition of gelatin alone and xanthan gum alone. The combination of these two alone enabled the emulsion to be dried without oil separation. 

Similar results were obtained by Vélez-Erazo et al. (2020) [30] where sunflower oil was structured also using an emulsion method with pea proteins and selected gelators, including xanthan gum. The system stabilized by xanthan gum was one of the steadier systems and had a creamy and smooth structure after homogenization. This emulsion was also characterized by lower weight loss during drying and no visible oil efflux. 

Xanthan gum together with gelatin was also used to produce oleogel using the foam method. In this case, studies confirmed the formation of a digestible foam, which, when freeze-dried, gives a porous cryogel that adsorbs oil well. The oleogel retained its structure at temperatures below 100 °C, making it suitable for food production at a maximum processing temperature of 100 °C. Studies have also shown that the oleogel network can retard oxidation and increase oil stability [28].

In all the cases presented above, xanthan gum formed stable oleogels containing more than 90% oil. Using both the emulsion and foam methods, the dried products had interesting microstructures. The oil droplets were tightly packed, and the biopolymer layer prevented coalescence of the oil and thus its separation from the structures. In two cases, these oleogels showed high gel strength and some degree of thixotropic regeneration (even at high temperatures) [16,28]. 

Vélez-Erazo et al. (2020) [30] confirmed that this system is stable for four weeks, which could lead to it being used in the future as a substitute for saturated fats in food products. However, research is still needed to assess the behavior of these oleogels in food products and to evaluate the release and oxidation of the oils under different storage conditions. 

### 3.2. Gellan Gum 

Gellan gum, like xanthan gum, is a biopolymer of bacterial origin with unique physicochemical properties. Gellan was discovered in 1978 by isolating the microorganisms that synthesize it from the tissues of Elodea plants. During the research, it turned out that aerobic non-disease bacteria (G-) of the genus *Pseudomonas*, originally named *Pseudomonas elodea*, were responsible for synthesizing this polysaccharide. The current name of these bacteria is *Sphingomonas elodea* [39]. It was initially used as a medium in microbiology laboratories, but now is widely used as a food additive all over the world. 

Gellan gum is an anionic linear polysaccharide with a very high molecular weight of 1000 g/mol. The basis of its structure is a repeating tetrasaccharide fragment consisting of β-D-glucuronic acid, α-L-rhamnose and β-D-glucose in a molar ratio of 2:1:1 [40,41], substituted with acyl groups (glyceryl and acetyl) as esters linked by O-glycosidic bonds [42]. Depending on the preparation method, two types of gellan gum can be distinguished: native (high-acyl gellan, HA gellan) and deacetylated (low-acyl gellan, LA gellan). 

Modifying the gellan molecule by controlling the number of acyl groups makes it possible to influence the rheological properties of the final product, such as its texture and viscosity, depending on its intended use. The native form has two types of substituent attached to the β-D-glucose residue. These are an acetyl group at the C-6 atom and an L-glycerol group at the C-2 atom [40]. During alkaline hydrolysis, a deacetylated form appears. Both forms swell in cold water and dissolve in hot water (80–90 °C). 

The polymer chains are then arranged chaotically, and the interactions between them weaken. During cooling, however, double helixes are formed, which aggregate to create a three-dimensional spatial network, resulting in a system with hydrogel properties. The highly acetylated form of the hydrogel shows a semi-solid consistency, is easily deformable, and its properties are similar to those of physical hydrogels. In contrast, the LA form forms elastic, hard and brittle gels that behave similarly to chemical hydrogels [43].

The structure of gellan gum is a polymeric network of relatively low porosity which resembles a spider web in appearance, in contrast to xanthan gum which resembles the structure of honeycomb. The pH of the solution, the presence of cations, and the concentration of gellan gum used, in addition to the appropriate temperature, have a significant influence on the mechanism of gellan gel formation. Due to the absence of cations, low-acyl gellan forms a gel at temperatures as low as 25 °C, whereas its high-acyl form gels at 65 °C. The gel forms quickly as soon as the appropriate temperature is reached. However, gelation with salt addition also occurs and is dependent on the type of salt added. 

The structure of gels in the presence of divalent ions is more complicated, in contrast to monovalent ions. The divalent cations contribute to the formation of successive aggregations of the double helix at lower temperatures than the conformational transformation of the gellan particles. In the case of divalent cations, a more ordered gel structure is formed at slightly higher temperatures than the conformational transformation of gellan gum. Gellan matrices are characterized by their sensitivity to pH. In acidic environments, they show stability, whereas as the pH increases, they absorb water and swell [44,45].

In the future, gellan gum can be used to shape and model the structure and texture of the matrix, which can be the building block of the biopolymer network in the formation of oleogels [30]. A small amount of gellan gum results in improved system stability and thus gelation temperature, which also translates into improved thermal stability of other gel products susceptible to melting under high ambient temperatures. 

Such an approach applies to vegetable, meat, and fish products. It is successfully used in producing jams, fruit fillings, jellies, sauces, dairy drinks, ice cream, yoghurts, or diet products. Thanks to its properties, it ensures good stability during the product’s processing, transport, and storage. Its characteristic gel structure and low solution concentration create products with both good taste and appearance. The ease of application in food products is desirable to both food manufacturers and consumers.

### 3.3. Alginates

Alginates are an example of polysaccharides of natural origin, which are increasingly used in the food and pharmaceutical industries due to their physicochemical and health properties. These are natural polymers that combine, unique among other biopolymers, chemical, biological, and physical properties such as biodegradability, biocompatibility, bioactivity, or membrane- and fiber-forming abilities [46]. 

They belong to a group of polyuronic saccharides extracted from marine algae, mainly brown algae (Phaeophyceae), or produced extracellularly by some bacteria such as *Azotobactervinelandii*, *Pseudomonas aeruginosa*, and *Pseudomonas fluorescens*. Alginates are also commercially extracted from such brown algae as *Ascophyllum*, *Laminaria* (Europe), *Lessonia* (South America), *Ecklonia* (South Africa), *Durvillaea* (Australia and Chile), *Macrocystis* (California), and *Sargassum* and *Turbinaria* as a source of lower quality alginates [47]. 

The extraction of alginates from brown algae involves extraction with a dilute alkaline solution to extract the alginic acid present in these algae. Free alginic acid is then extracted by treating the resulting viscous and dense mass with inorganic acids [46]. By hydrating the alginic acid, a high viscosity ‘‘acid gel’’ can be obtained. After the gelation process, water molecules are physically bound in the alginate matrix but are free enough to move. This feature is essential because of the potential use of alginate in many applications (e.g., alginate gels for cell immobilization/encapsulation) [47,48]. 

Alginates are polysaccharide copolymers that consist of β-D-mannuronic acid (M-blocks) and α-L-guluronic acid (G-blocks) residues linked together by glycosidic bonds. The L-guluronic block is in the 1C-4 conformation, and the D-mannuronic block is in the 4C-1 conformation, regardless of their nearest neighbor. The G and M blocks can occur in different proportions and in other distributions along the chain (possible distributions are GGGGGG, MMMMMM, GMGMGM) [46,49]. 

The molecular weight range is from 33,000 to 400,000 Da for commercially available alginates. Their structure depends on their origin (seasonal varieties, algal species, geographical origin), harvest time, extraction techniques, or the type of bacteria that synthesize them. The aforementioned conditions significantly influence the structural features of alginates, such as the arrangement of uronate residues, M/G block ratio, molecular weight, or degree of acetylation [50]. They also strongly influence physicochemical properties such as viscosity, sol/gel transitions, and water absorption [51].

Alginates rich in GG monomers have a higher solubility in water than those rich in MM. It has been shown that at low pH, alginates with more MG/GM monomer units are soluble, while alginates rich in GG or MM block are insoluble. The solubility and viscosity of alginates are also affected by the presence of acetyl groups. Gels formed by alginates with higher GG content are stronger and more brittle than those created with higher MM content. Stable and viscous solutions are obtained from esters of alginic acid. Solutions of alginates with long polymer chains (high molecular weight) tend to have a higher viscosity than those with shorter chains. Nonetheless, studies have reported that this correlation is not observed for alginates of bacterial origin [50]. Alginates derived from *Laminaria japonica*, *Ascophyllumnodosum*, and *Macrocystis* have been shown to have low G-block content, while alginates derived from *Laminaria hyperborea* have high G-block content [47]. 

Alginates, due to their excellent physicochemical properties, are successfully used to form oleogels. Wijaya et al. (2019) [17] used high molecular weight alginate (ALG) (350,000–500,000 Da) in combination with sodium caseinate (SC) protein to stabilize the structure of the resulting oleogel.

### 3.4. Pectines

Pectins may prove to be an interesting application of natural biopolymers for oleogel formation [30,52]. These are natural linear polymers, which are a complex mixture of sugars that include poly- and oligosaccharides. Pectins contain in their chain mainly α-1,4-D-galacturonic acid and saccharides such as d-galactose, l-fucose, d-xylose, l-arabinose, and l-rhamnose. The polymeric chain of pectins consists of 300 to 1000 monomers of α-1,4-D-galacturonic acid, linked to each other by α-1→4 glycosidic bonds. Some of the carboxyl groups of α-1,4-D-galacturonic acid are esterified with methyl groups [53]. 

Individual α-1,4-D-galacturonic acid fragments can be substituted with residues from other saccharides. Pectins have unique functional properties mainly due to their structure and origin. The highest amounts of these polysaccharides are extracted from citrus fruits (20–35% *m*/*m*), soybean hulls (25–30% *m*/*m*) [54], sunflower heads—seedless (15–24% *m*/*m*) [55], sugar beet (10–20% *m*/*m*) [56], mango peels (10–15% *m*/*m*) [57], and apple pomace (12% *m*/*m*) [58]. 

The most important polysaccharides building the structure of pectins are homogalacturonan, rhamnogalacturonan I, rhamnogalacturonan II, xylogalacturonan, arabinan, arabinogalactan I, and arabinogalactan. In pectin, these polysaccharides form alternately, thus forming regions consisting of linear, homogeneous chains (smooth region) made of α-1,4-D-galacturonic acid molecules linked together and branched, heterogeneous chains (hairy region) that form different structural polysaccharides [59]. The most abundant polysaccharide forming the structure of pectin macromolecules is homogalacturonan. It accounts for about 57–70% of the proportion of all pectin structural substances contained in plant cell walls [60]. 

For citrus, apple, and beet pectins, the polymeric backbone of homogalacturonan consists of at least 72–100 α-1,4-D-galacturonic acid molecules. As previously mentioned, some of the carboxyl groups located at C-6 in the α-1,4-D-galacturonic acid molecule can be esterified with methyl groups, and the degree of methylation ranges from 70 to 80%. The degree of methylation (DM) is one of the key parameters for pectins’ gelling ability. It is defined as the ratio of methyl esterified carboxyl groups to the total amount of α-1,4-D-galacturonic acid monomers and is determined by infrared spectroscopy.

The polymer chain fragments of homogalacturonan, consisting of 7 to 20 nonesterified parts of an α-1,4-D-galacturonic acid, show an enhanced ability to attach Ca^2+^ ions. Furthermore, homogalacturonan can also be partially O-acetylated at the second or third carbon atom. Homogalacturonan is soluble in water and alkaline medium, whereas it is hardly soluble in a slightly acidic medium. The second most abundant polysaccharide in pectin is rhamnogalacturonan I composed of at least 100 residues of α-1,4-D-galacturonic acid and L-rhamnose, alternately linked. Oligosaccharide units (e.g., D-galactose residues, L-arabinose and their derivatives) may be attached to the rhamnose fragments at the fourth carbon atom. 

The degree of branched rhamnose residues ranges from 20 to 80%, depending on the plant source and the pectin isolation method used. In the side chain of rhamnose, there are linear or branched α-L-arabinofuranosyl and β-D-galactopyranosyl residues. The side chain of rhamnogalacturonan I also contains ferulic and coumaric acid residues, as well as α-L-fucosyl, β-D-glucuronosyl and 4-O-methyl β-D-glucuronosyl residues. 

Acetyl groups may be present in the D-galacturonic acid residues at the second and third carbon atoms. Rhamnogalacturonan II consists of 12 different glycosyl residues that are linked to at least 20 different bonds. The polymeric backbone of rhamnogalacturonan II is built from 8 to 15 residues of partially esterified α-1,4-D-galacturonic acid and four side chains (at C2 and C3).

The glycosyl residues that build the side chains contain fragments derived from L-arabinose, D-galactose, L-rhamnose, and D-glucuronic acid, among others [59]. Similarly to rhamnogalacturonan I and rhamnogalacturonan II, xylogalacturonan is also a branched polysaccharide building pectin whose main polymer chain is based on α-1,4-D-galacturonic acid monomers, substituted most often at C-3 with β-D-xylose monomers. The degree of attachment of xylosyl fragments to D-galacturonic acid in the main chain depends on the plant origin and may range from 20 to 100%. 

Arabinogalactans are divided into types I and II, the skeleton I is built from β-D-galactopyranose residues connected by 1,4-glycosidic bonds, while type II is built from β-D-galactopyranose residues connected by 1,3-glycosidic bonds. In both types, strong branching of the main polymer chain is observed. The last polysaccharide building the structure of pectins and previously mentioned is arabinan. Its chain is composed of α-L-arabinofuranose residues linked together by 1→5-glycosidic bond. It usually contains single α-L-arabinofuranose residues glycosidically linked in a 1→3 or 1→2 manner and also double α-L-arabinofuranose residues attached by 1→3-glycosidic bond [59]. The technological classification of pectins is based on the degree of methylation (DM). It distinguishes between high methylated—HM (or highly esterified—HE) pectins in which more than 50% of the carboxyl groups of the galacturonic acid residues are esterified and low methylated—LM (or low esterified LE) pectins in which the degree of esterification is less than 50% [60]. 

Commercially available pectins are in the form of white, beige, or light brown powder and are used in many industries due to their gelling properties. The gelation process is influenced by temperature, pH environment, metal ion concentration, and polymer chain length resulting from the type of pectin used. Highly methylated pectins form gels at elevated temperatures and when their concentration is between 0.3–2.0%, at pH = 2.0–3.5 in the presence of at least 55% saccharides, most commonly fructose and sucrose [61]. 

Water molecules accumulate around larger saccharide molecules, thus allowing reactions between pectin chains and hydrogen bonds between hydroxyl groups. However, in an acidic environment, carboxyl groups are protonated, which decreases the electrostatic repulsion between the pectin chains, which have a negative electrical charge. The gelation process in pectin occurs mainly through hydrophobic interactions between methyl groups and hydrogen bonds between hydroxyl groups and non-dissociated carboxyl groups. Hydrogels obtained from highly esterified pectins are characterized by good softness and elasticity and do not exhibit syneresis (shrinkage by dispersion). In addition, they do not reveal the characteristics of regelatinization during heat treatment as they are thermally irreversible [59].

In contrast to high-methylated pectins, low-methylated pectins exhibit gelling capabilities at room temperature and pH = 2.0–6.0 and the presence of divalent metal ions (e.g., Ca^2+^). No addition of saccharides is required during the gelling process, as is the case with high-methylated pectins. Gelation occurs through the formation of ionic bonds between calcium ions and carboxyl groups in the pectin chains. The use of divalent ions causes the polysaccharide chains in pectin to align, which facilitates the binding of subsequent metal ions. In this way, the hydrogel formed from low-esterified pectins shows thermal reversibility so that after heating, melting, and cooling again, these pectins can gel again [59]. The attractiveness of pectins due to their favourable gelling properties is successfully used to form a biopolymer network that creates a matrix for oleogels. An example of the use of pectins to obtain oleogels is the work of Luo et al. (2017) where highly esterified citrus pectin (DM > 65%) was used.

### 3.5. Tara Gum and Carob Gum

Tara gum and carob gum (locust bean gum) have similar origins as both are derived from the seeds of pod trees [62]. Both these gums belong to a group of polysaccharides called galactomannans. They are polysaccharides of plant origin having the main polymer chain consisting of ß-D-mannopyranose units linked by ß-bonds (1→4), which are substituted irregularly by glucose linked by α-bonds (1→6). The arrangement of the side chains depends on the source of origin. They are non-gelling hydrocolloids, but give solutions with high viscosity [63]. 

The main component of tara gum is linear chains of ß-D-mannopyranose monomers with α-D-galactopyranose units attached through bonds (1→6). In tara gum, the ratio of mannose to galactose is 3:1, while in carob gum, the ratio is 4:1.

This feature of tara gum provides an opportunity for interesting rheological characteristics and industrial applications, since the solubility, rheological, and other functional properties of galactomannans are strongly dependent on the molar mass, mannose/galactose ratio and also on the distribution of α-D-galactopyranose along the main biopolymer chain [64]. 

The molecular weight of tara gum is also high at around 1,000,000 Da [65]. Both tara and carob gums form solutions characterized by high viscosity at low shear rates and strong shear thinning [66]. 

The rheological properties of tara gum are influenced by concentration, pH, temperature, presence of salt, and sucrose. Studies obtained by Wu et al. (2015) [67] prove that tara gum exhibits non-Newtonian, pseudo plastic behavior in concentration ranges of 0.2–1.0%. However, the addition of CaCl_2_ and NaCl salts caused a decrease in its viscosity, where the drop was more pronounced for Ca^2+^ ions than for Na^+^. The gum also showed stable viscosity over a wide pH range (pH 3–11), and the effect of sucrose depended on the concentration of the biopolymer. Additionally, increasing the temperature from 20 °C to 80 °C reduced the viscosity of the gum. Frequency sweeps showed that for a concentration of 1% (*w*/*v*), tara gum behaves like a liquid at low frequency and a gel at high frequency. The viscous properties of the gum also increase with increasing concentration [68]. The molecular weight of tara gum is 50,000–3,000,000 Da [67]. 

Carob gum, otherwise known as locust bean gum, is partially soluble in cold water and therefore requires heating for complete dissolution, but its solubility never exceeds 90% in heated solution. In the pH range of 4–9, the viscosity of carob gum solution is stable, but decreases with pH above 9 and below 4. Carob gum is resistant to mechanical deformation, but its rheological properties depend on molecular weight, concentration, shear rate, or solubilization method. However, this gum is often considered less viscous than tara gum. Carob gum in aqueous solutions exhibits non-Newtonian behavior (pseudoplastic) at high shear rates, but shows Newtonian flow properties at low shear rates. 

At lower shear rates, the disruption of entangled particles can be counterbalanced by the reformation of new entanglements so that the viscosity is constant. At higher shear rates, the disruption of nets dominates the formation of new tangles of particles that align in the flow direction, leading to a reduction in viscosity and thus explaining the non-Newtonian behavior of carob gum at higher shear rates. Studies have confirmed that aqueous solutions of carob gum with concentrations ranging from 0.5% to 2.0% exhibit liquid-like behavior at lower frequencies (G″ > G″) and solid-like behavior at higher frequencies (G′ > G″) [69]. 

Both tara gum and carob gum have been successfully used to form oleogels due to their viscous properties [30].

### 3.6. Carrageenan

Carrageenan is one of the hydrocolloids commonly used in food production and thus a polysaccharide with considerable potential as an oleogelator is carrageenan [30,70,71]. This polysaccharide, along with agar, consist a part of the cell walls of seaweeds and constitute 40–50% of the dry weight of these algae. Carrageenan due to its biocompatibility, biodegradability, and low toxicity is often used in pharmaceutical, food, and cosmetic industries [71,72]. Carrageenans are obtained by extraction from several species of red seaweeds (Rhodophyta) such as *Eucheuma*, *Gigartina*, *Hypnea*, *Kappaphyccus*, *Chondrus,* and *Mastocarpus*. 

There are currently six basic forms of carrageenans and these are: (kappa) κ-, (lambda) λ-, (mu) μ-, (jota) ι-, (nu) ν-, and (theta) θ-carrageenan [73]. The type of form obtained depends on the extraction method and the species of seaweed. The most common fractions are kappa (κ-), lambda (λ), and iota (ι) [74]. The classification of carrageenan was developed based on its solubility in potassium chloride. 

Carrageenan is a polygalactan, characterized by having a variable number of sulfuric acid (VI) residues. The main polymeric chain of carrageenan consists of 3-6-dehydro-D-galactose and D-galactose molecules which are connected by α-1,3 and β-1,4 glycosidic bonds [75]. The basic differences affecting the properties of carrageenan, which thus define its form, are the number and position of sulphate groups (VI) and the number of 3,6-dehydro-D-galactose monomer molecules in the main chain. 

Studies show that the higher the content of sulphuric acid (VI) residues in the polymer, the lower the dissolution temperature; however, carrageenan hydrogel is characterised by lower resistance to external factors. Kappa carrageenan has a sulphate group content of about 25% to 30% and a 3,6-dehydro-D-galactose content of about 28% to 35%, while lambda carrageenan has a sulphate group content of about 32% to 39% and no 3,6-dehydro-D-galactose. 

Sulphate groups, which are strongly anionic in nature, also affect the reactivity of carrageenan. For this reason, their reactivity is comparable to their counterparts in the form of inorganic salts. This property of carrageenan translates significantly into its stability, which is why only its sodium/potassium and calcium salts or their mixtures can be found commercially. This is also reflected in the physical properties of carrageenan, which are influenced by the associated metal cations with the conformation of the sugar monomer units in the main chain. The iota and kappa forms creates gels in the presence of potassium and calcium ions, whereas the lambda form does not possess the ability [73,75,76]. 

Studies conclude that the functionality of carrageenans largely depends on their rheological properties. Carrageenan is a linear, soluble polymer and generally forms viscous solutions. Its viscosity depends on temperature, concentration, carrageenan type, molecular weight, and the presence of other solutes. Studies report that carrageenan viscosity increases exponentially with concentration and decreases with temperature. Carrageenans are sensitive to depolymerisation by acid catalysed hydrolysis, which at low pH and high temperature results in loss of carrageenan functionality. The molecular weight of commercial carrageenan ranges from 100,000 to 1,000,000 Da [73,75,76]. 

As mentioned earlier, carrageenan has been successfully used as an oleogelator to form oleogels. Vélez-Erazo et al. (2020) [30] obtained the most stable oleogels with carrageenan (with the iota form predominating in the composition). The oleogel with carrageenan showed no difference during the analysis time (28 days), which allowed the researchers to conclude that it could be used as a substitute for saturated fats in the future, as it is highly stable during storage [30].

### 3.7. Agar

An interesting polysaccharide that may in future be used to structure oils and thus form oleogels is agar. This polysaccharide is obtained from kelp from the Gelidiaceae and Gigartinaceae families. It consists of two monomers: agarose and agaropectin. Agaropectin contains molecules of D-galactose and galacturonic acid, to which a variable number of sulfate (VI) residues are attached, while agarose is an unbranched polysaccharide formed by molecules of α-(1→4)-3,6-dihydro-L-galactose and β-(1→3)-D-galactose [75,77]. 

Due to the attached sulfuric acid (VI) residues, calcium, magnesium, potassium, and sodium cations bind to agar [78]. Depending on the preparation method, agar occurs as powder, scales, long flat light yellow or transparent streaks or grey lumps with a porous structure. Its color depends on its purity and varies from pale yellow through yellow-grey to orange, while its aroma, due to the presence of traces of additional substances, maybe reminiscent of the marine smell. When exposed to cold or hot water, agar swells to form a viscous, gelatinous colloidal solution which, when cooled to about 35–50 °C, solidifies to form a gel.

For this reason, it is widely used in the cosmetics industry for the production of fat-free creams and masks as it improves the lubricity and adhesion of these preparations [79]. Agar is also used as a thickener, stabilizer, and emulsifier. This polysaccharide is commonly used in the food industry as a substitute for animal gelatin. It is a natural and tasteless gelling agent (E406) used, for example, in the manufacture of confectionery (jams, jellies). Its thickening and gelling properties are also used to produce unfermented milk drinks, salad dressings, and pastry products, etc. Agar gels have long been used to formulate food and pharmaceutical products and have been accepted by manufacturers as well as consumers. However, there is still a lack of literature reports on the use of agar to form oleogels. 

Kodela et al. (2017) [80], in their study, successfully obtained biogels using agar gel as the aqueous phase and oleogels based on rice oil and stearic alcohol as the non-polar phase. Microscopic examination of the products obtained suggested that the introduction of oleogels into the agar hydrogel resulted in the formation of structures in which the oleogels were present in the form of spherical particles in the continuous phase of the agar hydrogel. 

On the other hand, studies of mechanical properties showed finding a limiting concentration of oleosol in the hydrogel after which these properties deteriorate [80] and the cosmetics industry for the production of fat-free creams and masks, as it improves the lubricity and adhesion of these preparations [79]. 

### 3.8. Chitin 

An interesting future use of a polysaccharide as an oleogelator could be chitin. Chitin is the most abundant naturally occurring neutral polymer after cellulose. The main sources of chitin are shells of crustaceans such as crabs, shrimps, lobsters, and krill. Another large source are fungi, where chitin is found in their cell walls as one of the main fibrous polymers. Chitin is biodegradable, biocompatible, accelerates wound healing, and enhances immunity [46,81]. It is a natural polymer that is composed of N-acetyl-D-glucosamine molecules linked by a β-1,4 glycosidic bond. The molecular weight of chitin is ≤1,000,000 Da. 

Chitin has a highly ordered crystal structure due to the presence of many hydrogen bonds. It has polymorphic α, β, and γ structures, which differ in the spatial orientation of the polymer chains, which affects its hardness and solubility, since virtually all of its forms show insolubility in solvents. This is one of the reasons that hinders its applicability. For this reason, research is being conducted on chitin derivatives, which, while retaining its biological parameters, show better physicochemical properties, thus enabling their application. 

Chitosan is an example of chitin modification. It is deacetylated chitin obtained by chemical or enzymatic deacetylation process. Chitosan is used in the field of biology and medicine because of its biodegradability, non-toxicity, biocompatibility and anticancer and antibacterial properties. Its disadvantage, unfortunately, is that it only dissolves in certain dilute acids, which makes its application much more difficult. However, its molecular structure opens up many possibilities for its modification due to a large number of active functional groups, thanks to which it is possible to create derivatives with improved chemical, physical, and physiological properties. 

An example of the use of crude chitin is the work of Nikiforidis and Scholten (2015) [82]. In this experiment, it was shown that chitin can have some oleogelating potential, however, with the participation of surfactants because mixing chitin and oil alone caused too much aggregation of the organogelator (chitin) resulting most probably from its difficult solubility [82]. 

Currently, chitin can be used in the form of its derivatives, which are obtained during chitin modification. An example of the use of a chitin derivative, more specifically regenerated chitin, is the research work of Baraki et al. (2021) who, using this type of oleogelators as stabilising particles, obtained stable oleogels that had good stability when heated to 80 °C for 2 days [83]. However, there are still few literature reports using chitin or its derivatives as the main gelling agents.

## 4. Physicochemical Properties of Polysaccharide-Stabilized Oleogels

Structuring liquid oil with polysaccharide biopolymers results in potential products with low and high polyunsaturated fatty acid content. Depending on the properties of the applied biopolymers (oleogelators), conditions, and method of preparation, final products with desired physicochemical, rheological, and organoleptic properties can be obtained. The polysaccharide biopolymers mentioned above are characterized by interesting properties such as emulsifying, stabilizing, or gelling properties. The scientific community recognized the structuring of oils and fats using polysaccharides as a potential alternative for developing new products with improved nutritional profiles (Table 1). 

Vélez-Erazo et al. (2020) [30] using emulsion oleogelation with pea protein as an emulsifier and various polysaccharides (carrageenan, acacia gum, sodium alginate, pectin, gellan gum, tara gum, carob gum) as stabilizers, obtained high internal phase emulsions (HIPEs) characterized by a highly concentrated volume of the internal phase (dispersed phase). The share of the dispersed phase in such an emulsion is 0.74, which causes the deformation of its droplets into polyhedrons, which are separated from each other by thin layers of the continuous phase. 

The structure of these emulsions is analogous to a standard gas–liquid foam with low liquid content and interesting properties, including high viscosity and interesting viscoelastic behavior [84]. In this study, oil-in-water 60/40 emulsions were prepared at a protein: polysaccharide ratio of 4: 1 and the samples were then dried in an oven to obtain HIPE [30]. 

The obtained HIPE samples were subjected to interfacial tension, macro- and microstructure, droplet size, water and oil loss and rheological analysis. The interfacial tension, in this case, was used to investigate the dynamics of protein adsorption on the oil/water interphase upon emulsion formation. All mixtures showed an induction period lasting 30 s where the interfacial tension did not decrease, followed by a voltage spike lasting 1000 s when the protein regroups at the interface. In the last stage, where a pseudo equilibrium is observed, the emulsions showed voltages between 6 and 10 mN/m except for the pea protein: gum arabic and pea protein: gum tare systems, where the values were 25 and 19 mN/m, respectively, which may suggest that the addition of protein caused an increase in interfacial tension [30].

All emulsions were subjected to mass balance studies after 48 h; however, already after 24 h, visible phase separation was observed for the pea protein: gum arabic system, probably due to the low viscosity of gum arabic in comparison with the other polysaccharides used. On the other hand, for the pea protein: xanthan gum and pea protein: tara gum systems, a soft structure was observed for the dry products and a stable structure for the other systems. After homogenization, the samples showed different behavior. The pea protein: acacia gum and pea protein: guar gum systems showed the highest and most significant oil release and water loss after 48 h of drying (37.68 and 37.11%, respectively) [30]. 

This characteristic could most likely be related to the low water retention capacity of these polysaccharides in the system, which destabilized the complex matrix observed in the product before homogenization, causing a clear phase separation after homogenization of the sample. In the other systems, oil separation after homogenization was lower. The most stable systems were pea protein: xanthan gum and pea protein: tara gum, which had the lowest weight loss after drying (22.51% and 18.87%, respectively) and no visible oil release after homogenization. Their consistency after homogenization was very promising and resembled a “mayonnaise” type structure [30]. 

This type of HIPE is comparable to the systems described by Wijaya et al. (2017) [29], who obtained oil emulsions stabilized by complexes of pectin and whey protein isolate. 

The rheological analysis revealed that none of the systems showed thixotropic properties. All emulsions exhibited shear-thinning behavior (n between 0.49 and 0.62), and pea protein: xanthan gum, pea protein: carob gum and pea protein: carrageenan mixtures showed initial shear stress of 10.02, 3.18 and 2.01 Pa, respectively, which may indicate that such mixes require an initial force for the structure to start flowing. 

The stabilizing ability of polysaccharides was also observed in the viscosity correlation study as the emulsion droplet size decreased. The higher the viscosity capacity of the polysaccharides (tara gum and xanthan gum), the lower the movement and coalescence of the emulsion droplets and thus the smaller the droplet size. This phenomenon is explained by the pea protein: acacia gum system, the largest droplet size was observed due to flocculation and coalescence of emulsion droplets not fully coated with pea protein. 

Based on the value of G′ and the difference between G′ and G″ and the linear length of the curves, the strength of the formed gel was evaluated. For low-stress values, the G′ value of the pea protein: xanthan gum emulsion was similar to that of the pea protein: guar gum system. It was about 135 Pa, thus showing higher elasticity than the pea protein: tara gum and pea protein: carob gum systems, which presented G′~ 65 Pa. In the case of HIPE, a significant increase in G′ was evident, amounting to 580 Pa for pea protein: tara gum and 830 Pa for pea protein: xanthan gum.

These systems also maintained the linearity of the curve at high stresses, especially HIPE pea protein: xanthan gum, which had the highest elasticity characteristics. The frequency sweep tests confirmed the results obtained during the stress sweeps at 0.1 Pa. Both emulsions and HIPE showed gel-like behavior as the G′ modulus was more significant than the G″ modulus. In the frequency sweeps, it was observed that all systems (emulsions and HIPEs) had good strain tolerance due to the positive angular factor G′ of the structure and the small dependence of G′ on frequency. The reliance of emulsion and HIPE sensitivity on thermal transformations was also an important observation. The emulsions and HIPE’s gel-like structures (G′ > G″) did not change under temperature changes, suggesting that all samples are thermostable. Similar results for oscillating stress, frequency, and temperature sweep were obtained by other researchers for emulsions containing 60–75% oil and HIPEs also obtained by emulsion drying [33,85,86,87,88]. 

In the experimental work of Vélez-Erazo et al. (2020), [30] the pH of the biopolymers was not modified, and the polysaccharides were added after emulsifying the oil with the protein, obtaining an emulsion with pH ~6.0. The contribution of the polysaccharides’ steric properties and the pea protein’s interfacial properties is more significant for stabilizing the system than electrostatic interactions when the strength of electrostatic interactions at pH 6.0 is reduced [30]. 

An example of work where the effect of pH and protein to polysaccharide ratio on the properties of the resulting oleogels was investigated is the experiment performed by Wijaya et al. (2019) [17]. In this study, a series of aqueous solutions with SC: ALG ratios of 6:1, 8:1, 10:1, and 12:1 were prepared and brought to three pH values of 5.5, 6.0, and 7.0 such that each pH range corresponding to each solution with given protein to alginate ratios, thus obtaining a total of 12 solutions. In parallel to these solutions, one series of solutions of SC: ALG conjugates were obtained in proportions of 6:1, 8:1, 10:1, and 12:1 only for pH = 7. These were first lyophilized, then heat-treated in an oven at 60 °C for 2 days at a relative humidity of about 74% before being dissolved in demineralised water to obtain solutions of similar dispersion to those described above. These solutions were then mixed with oil at a volume ratio of oil:protein–alginate aqueous solution (80:20 *v*/*v*) and homogenized to give high internal phase emulsions (HIPEs) [17].

**Table 1:** The process conditions for manufacturing of natural gum based oleogels.

Oleogelator	Oil	Type	Conditions	Ref.
sodium caseinate (SC) and alginate (ALG)	SUNFLOWER OIL	EMULSION	Oleogels were made according to two schemes: 1.SC:ALG aqueous solutions and 2. SC:ALG after dry heat treatment (DHT), so-called conjugates. SC and ALG powders were dissolved separately in demineralised water containing 0.02 % sodium azide to obtain the concentration of 12% and 1%, respectively. The dispersion were stirred continuously at RT until complete dissolution. The solutions were stored at 5 °C to ensure complete hydration of the biopolymers. To study the effect of SC:ALG ratio, solutions were prepared in ratios of 6:1, 8:1, 10:1, and 12:1 by appropriate dilutions of stock solutions and brought to three pH levels of 5.5, 6.0, and 7.0 each with 1,0 M HCl and 0,1 M NaOH. The solutions were ultrasonicated for 30 s using a head sonifier. To compare the mixture and conjugates formed by DHT, the first step was repeated but only for pH 7. The frozen samples were lyophilized for 48 h and transferred to a desiccator containing saturated NaCl solution and placed in an oven at 60 °C for 2 days at RH of 74%. After DHT, the SC:ALG conjugates were dissolved in demineralised water to give a series of dispersion systems as described in the first step. Then oleogels were formed by mixing sunflower and solutions obtained from the first and second preparation in a volume ratio (80:20 *o*/*w*). The mixtures were homogenised using a head homogenizer first at 6500 rpm and then at 9500 rpm for 2 min.	[17]
pea protein (PP), carrageenan (CG), pectin (PC), xanthan gum (XG), gellan gum (GG), acacia gum (GA), sodium alginate (AL), locust bean gum (LBG)and tara gum (TG).			The dispersion (2.0% *w*/*w*) systems of PP and eight types of polysaccharides: CG, XG, GA, AL, PC, GG, LBG, and TG were prepared using Milli-Q water. The solutions were stirred at RT and then left overnight for complete hydration of the biopolymer. Oil-in-water emulsions (60/40) were formed by mixing sunflower oil with an aqueous PP solution using a head homogenizer at 5500 rpm for 6 min. Then, the polysaccharide solution was added to the emulsion in a ratio of 4:1 (emulsion/polysaccharide) and the emulsion was homogenised again at 11,000 rpm for 2 min. The resulting emulsion was dehydrated in an oven at 65 °C for 48 h. The product thus obtained was homogenized manually and then water loss was studied by gravimetric method.	[30]
gelatine, xanthan gum			Stock solutions of gelatine and xanthan gum were prepared in distilled water. The emulsion of oil dispersed in gelatine solution was prepared using a head homogenizer at 11,000 rpm and then xanthan gum solution was added. The procedure was repeated to form an emulsion in which the oil was first dispersed in the xanthan gum solution and then the gelatine solution was added. Drying of the emulsion was carried out in two ways. In the first one, standard oven drying at high temperature (70 °C) was used, for 48 h, while in the second approach freeze-drying using a freeze-dryer during which the samples were frozen to −23 °C and then dried under vacuum for 72 h during the sublimation process. In this way, oleogel systems containing more than 97% by weight of oil entrapped in the matrix were obtained. The formed oleogels can be successfully carried back into aqueous solutions by adding a calculated amount of water and homogenizing.	[16]
elatine nanocomplexes GLT), tannic acid (TA), linseed gum (FG)	SOY OIL	EMULSION	18 solutions of gelatine and tannic acid were prepared in distilled water. The pH of the solutions was brought to 6,0 with 1 M HCl or NaOH. Colloidal complexes were then produced by adding TA solutions to GLT solutions in mass ratios of 1:4, 1:8 and 1:16 while stirring for 1 h. The final solutions contained 1.2 wt % gelatine and 0.075–0.3 wt % tannic acid. Linseed gum stock solutions were prepared, then added to the GE and GE–TA solutions and stirred for 3 h to form complexes. The final GLT–TA–FG solution contained 1.2% *w*/*w*. GLT, 0.075% *w*/*w*. TA and 0.6% *w*/*w*. FG. The particle size and zeta potential of the colloidal complexes were measured at RT. Colloidal complexes and oil (45 wt %) were emulsified using a homogenizer at 12,000 rpm for 2 min to form an emulsion; 30 mL of the emulsion was poured into a glass Petri dish or plastic bottle 1.5 cm and 4 cm thick for different batches, respectively, and the samples were lyophilized. Part of the sample was oven-dried (60 °C) in a 100 mL beaker for 48 h until a solid mass was reached. To obtain subsequently soft oleogels, samples were homogenized at 10,000 rpm for 2 min.	[34]
citrus pectins, tea polyphenol palmitate	CAMELLIA OIL	EMULSION	Oleogels were formed using solutions with concentrations of 2.5% (*w*/*v*) of tea polyphenol palmitate and citrus pectin solutions with variable concentrations of 1.5; 2.5; 3.5, and 4.5% (*w*/*v*). Dispersion of oil in a 2.5% (*w*/*v*) solution of tea polyphenol palmitate was achieved by flash cooling using liquid nitrogen. The dispersion was carried out until the palmitate clearly stood in camellia oil, and then recrystallization of the palmitate was carried out by flash cooling in liquid nitrogen. Citrus pectin solutions of 1.5–4.5% (*w*/*v*) were intensively stirred of 800 rpm/min. at RT temperature. Then, the prepared emulsions (*o*/*w*) in amounts of 60 mL were mixed thoroughly with 40 mL of distilled water using a high-speed emulsifier at 8 ± 2 °C and 20,000 rpm for 2 min. The resulting emulsions were dried lyophilized for 48 h to remove the aqueous phase. The finished oleogels were obtained by shearing the freeze-dried products at 10,000 r/min for 2 min.	[52]
gelatine, xanthan gum		FOAM	Six gelatine and xanthan gum solutions were prepared by dissolving in distilled water. Equal volumes of all six solutions were then aerated using a homogenizer at 13,000 rpm for 5 min. to produce an aqueous foam solution. The foams were then frozen at −20 °C overnight, and lyophilized for 24 h to form a solid cryogel. A certain amount of oil was then added until the sample was saturated. The product thus prepared was sheared for 0.5–2 min at 10,000 rpm to obtain an oleogel.	[28]

The emulsions obtained in this way were studied later to determine the volume-weighted average droplet size (D4,3), microstructure, and stability. A significant decrease in droplet size and tightly packed oil droplets was observed with increasing pH value and SC to ALG ratio. HIPE emulsions prepared using SC: ALG mixture at pH 7.0 and SC: ALG conjugates showed the appearance of the finest oil droplets at SC: ALG ratios of 10:1 and 12:1. However, it should be noted that even at the lowest ratio, i.e., 6:1 and pH, the emulsion showed good stability without oil coalescence. This is most likely related to the synergistic effect of the protein and polysaccharides. Previous studies show that stabilization with protein alone did not give satisfactory results compared to systems where polysaccharide was additionally used. The average droplet size in the emulsions ranged from about 6.4 to 13.8 nm. Most likely, the increase in protein relative to the fixed compactness of alginate resulted in greater availability of its surface area for the adsorption of fat droplets and thus reducing the oil droplet size in HIPEs [89].

Furthermore, pH strongly affected droplet size; this is related to sodium caseinate reaching the isoelectric point pI (i.e., 3.8–5.8) [90] and its lower surface activity. This is due to a pronounced electrostatic complexation which, as a result of masking of hydrophobic casein groups by adsorbed polysaccharides, prevented oil adsorption on the protein surface, and thus resulted in an increase in droplet size with a decrease in pH to 5.5. Similar results concerning the effect of pH on the adsorption and interaction of SC at the interface with the polysaccharide (linseed gum) and thus on droplet size was obtained by Zhao et al. (2015) [91].

Oleogels obtained after drying and homogenization of HIPE was then imaged by scanning electron cryomicroscopy (CryoSEM). HIPE and oleogel samples were imaged for SC: ALG ratios of 12:1 at different pH values and for conjugates to determine the microstructure. The microstructure studies confirmed previous reports in which oleogels stabilized with biopolymer complexes exhibited a plastid-mid structure [92]. In addition, it was found that no oil escape (leakage) was observed from systems obtained from emulsions and conjugates with SC: ALG ratios of 10:1 even after two months of storage. Furthermore, it was also found that the presence of stiffened boundary surfaces at pH 7.0 did not cause coalescence of oil droplets giving a soft gel in which the microstructure was retained even after homogenization (shearing). The oleogel retained a homogeneous continuous structure without any visible deoiling (de-greasing), suggesting evenly distributed oil droplets in the biopolymer network. 

As mentioned above, oleogels obtained from alginate exhibit interesting viscoelastic properties. To determine the rheology of obtained HIPE emulsions and oleogels, amplitude stress and frequency sweeps were applied. These tests allowed to establish a linear viscoelastic region (LVR) for HIPE and oleogels. The gel strength was strongly correlated with the dynamic yield stress, and the critical stress, i.e., the point where G″ crosses G′ and a change in viscoelastic behavior occurs during which the substance starts to flow due to damage to the internal structure. When tested by sweeping the stress amplitude, all the samples showed a stable structure and did not change into the fluid at relatively high stress.

Furthermore, all samples showed constant behavior (i.e., higher G′ than G″ in the viscoelastic region) with G′ values around 8488 ± 221–72,793 ± 3181 for oleogel samples and above 995 ± 1–2158 ± 15 Pa for HIPE samples. The oleogel samples obtained from HIPE emulsions with pH 5.5 showed significant differences for G′ compared to oleogels obtained from emulsions with higher pH. Similarly, the HIPE emulsions themselves showed a higher value for G′, but a lower value for yield stress. This is due to the poor emulsifying properties that result from the adsorption of complex particles on the surface of oil droplets. The adsorption of complexes with larger sizes compared to SC and ALG particles resulted in a non-compact structure, which affected the stability of the oleogels after drying and shearing (second homogenisation).

## 5. Food Applications of Oleogels

**Figure 4:**
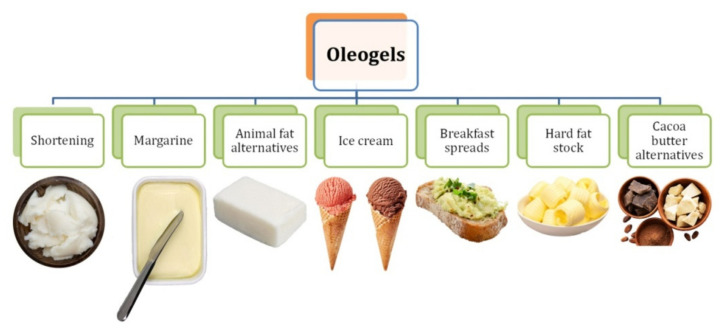
Products dedicated for possible oleogel incorporation.

The growing interest in oleogelation is constantly observed [10]. Oleogels may in the future be used in the food, pharmaceutical, cosmetic, and petrochemical industries [93]. One of the possible applications in the food industry that has been previously mentioned is undoubtedly to reduce the use of solid fats rich in saturated fatty acids and trans isomers and also to minimise the migration of edible oils in multi-ingredient food products [94] (Figure 4).

The oleogels role in meat products would be to partially replace beef fat, with which they share a similarity in fat particle size [3]. Depending on the biopolymer used, which determines the following properties of the oleogel obtained. They can be used as fatty ingredients in various food products. In all likelihood, structured edible oils will find a variety of industrial applications in the future and will thus draw even more interest from the scientific community in this topic.

Oil emulsions could become an alternative solution in pastry or confectionery products, and the health benefits of avoiding the harmful trans fats will increase consumer interest. One recent study shows that using oleogels based on natural biopolymers such as pectin has a positive effect on confectionery and proved that adding 50% (*v*/*v*) of the dispersed hydrogel to the other chocolate ingredients produced a low-fat chocolate product resistant to melting at 80 °C [95]. Previous studies on applications and potential uses in food products presented results in which oleogels based primarily on structures using oleogelators such as ethylcellulose were used [96,97].

In the pastry industry, oleogels could substitute so-called shortening agents, which contain animal fats in their composition. Luo et al. (2019) demonstrated that adding oleogels consisting of lemon pectin and oil to dough resulted in fewer bubbles during baking than those where traditional butter was used, most likely due to the absence of fat crystals and emulsifiers in the oleogels.

Additionally, it was found that as the pectin concentration in the oleogel matrix increased, the number of air bubbles in the dough decreased. This is a consequence of the dough’s mechanical strength, which prevents the introduction of air during the mixing process. The internal structure became firmer and more compact when the pectin concentration in the oleogels was increased to 4.5% (*m*/*v*). The samples for which the pectin concentration in oleogels was 1.5% and 2.5% (*m*/*v*) showed good dough quality [52].

Texture analysis and sensory evaluation of the cakes showed that the hardness of the baked goods where oleogels were used is higher than in those where butter was used, without a significant difference in elasticity in all samples and is similar to the results obtained by other researchers [88,98]. This was due to the dough structure produced in which pectin formed a network, further increasing the hardness of the baked goods [52].

Another interesting finding was that all baked goods samples, both where butter and oleogels were used, did not differ in aroma. Replacing solid fats with oleogels in baked goods seems on the surface to be a simple undertaking that would reduce saturated fatty acid content. However, formulating food products without the addition of solid fats is quite a difficult task. These are primarily responsible for the required structure, texture and, above all, taste sensation. Therefore, partial replacement of butter with oleogels is a promising method to reduce saturated fatty acids and trans fatty acids while maintaining the desired physical properties of baked goods [52].

The above-presented research using more natural polysaccharides, such as, e.g., alginates and pectins, is a novel endeavor and opens new insights into applications using such oleogels in low-fat foods [52,95]. Additionally, in the future, these oleogels may find broader application in food and respond to increasingly demanding consumers and food manufacturers promoting healthy and organic nutrition.

## 6. Conclusions and Future Remarks

Recent changes in consumer awareness and food producers trying to meet the needs of their customers have resulted in the introduction of innovative formulations in food products in response to market demands. One of such formulation is oleogel—solid fat form structured by using natural biopolymers.

In the future, oleogels may eliminate saturated and trans fats from food products and allow the production of low-fat products, thus reducing the environmental damage caused by the excessive use of palm oil in food. In addition, the increasing fictionalization of food forces producers to look for new solutions and improve their products to attract more conscious consumers who pay more attention to the food they buy. Innovative solutions for using oleogels based on biopolymers would be to introduce them into the food matrix and replace fats completely or to create blends containing the original source of fats and the addition of the oleogel.

Currently, there are no commercially available food products containing oleogels, despite the promising results of oleogel methods, the availability of natural oleogelators, and the similarities in physical properties between products based on conventional fat made with oleogels. The use of oleogel techniques in food technology is also supported by the positive results of sensory analysis of products containing oleogels.

However, there is still a need for new, efficient, and inexpensive oleogelators with specific processing conditions. Hence, there is a great need to investigate further the effects of various specific processing parameters on the properties of oleogel-containing foodstuffs. The most promising gelators in this field seem to be natural polysaccharide gums, which, in a possible (potential) application in food products in the future, will not raise negative feelings among consumers due to their natural origin.

However, there are still few literature reports and studies conducted on oil-structuring polysaccharides. The current pressure on the food industry to transform its products and adapt them to new health and sustainability trends will increase the importance and interest in oil structuring methods in the future, which will provide more knowledge in this field.

## Data Availability

Not applicable.
